# Co-creation to Develop Interventions to Facilitate Deep Reflection for Dental Students

**DOI:** 10.5334/pme.16

**Published:** 2023-03-16

**Authors:** Faith Campbell, Nicole Hassoon, Khalil Jiwa, Julia Ridsdill-Smith, Amie Smith, Helen Wilson, Kirsten Jack, Helen Rogers

**Affiliations:** 1 Academic Unit of Oral Health, Dentistry and Society, School of Clinical Dentistry, University of Sheffield, UK; 2 Academic Unit of Oral Health, Dentistry and Society, School of Clinical Dentistry, University of Sheffield, UK; 3 Faculty of Health Psychology and Social Care, Manchester Metropolitan University, UK; 4 School of Dental Sciences, Newcastle University, Framlington Place Newcastle upon Tyne, UK

## Abstract

**Background:**

Deep reflective practice is important in healthcare education to allow students to explore emotions associated with the learning experience, access deeper learning and develop their personal and professional identity. Previous research demonstrated that the current methods of reflective practice using logbooks at the end of a clinical session to facilitate reflection within this setting were viewed as suboptimal by staff and student users. To address this problem co-creation, or a ‘students as partners’ approach, was used to develop and implement a comprehensive intervention to facilitate deep reflection for undergraduate dental students. This included the production of educational resources, and development of an online safe space to reflect.

**Approach:**

In this paper we discuss the process of using co-creation with undergraduate dental students as a research methodology to successfully produce curricular change with respect to reflective practice by involving the voice and experience of student partners. These student partners were part of a team that included researchers and teaching staff and worked with other stakeholders within the institution within a wider team.

**Evaluation:**

This paper demonstrates the positive benefits of using co-creation with undergraduate dental students for students such as increased confidence, developing professional and personal skills and impacting meaningful change.

**Reflection:**

For researchers and educators, the process gave motivation and enjoyment in curricular development to address pedagogical problems and ensured that the developed intervention was sustainable and appropriate. The paper discusses benefits and challenges of co-creation to develop curricular change. This co-creation approach is recommended for solving similar problems in healthcare education.

## Background & Need for Innovation

Reflection is essential to support the personal and professional development of healthcare students. Reflective practice can help learners to bridge the gap between theory and practice, allowing them to find answers that they are unable to access through formal learning, whilst exploring the emotions associated with their learning experience [1]. In healthcare, reflection uses authentic, experiential activities, to elicit a deeper form of learning, allowing the generation of ‘transformative knowledge’; new knowledge that compels the clinician to change their practice behaviour [2]. This is particularly relevant in a healthcare setting where learners may feel anxious or insecure regarding their role and contribution to patient care and these negative emotions can impede learning [3].

Reflection is a requirement for dental professionals, as stated by numerous regulators globally [4,5,6], and is essential for undergraduate students throughout their dental education [7]. Reflection can help students to develop professional identity, self-confidence, alongside challenging assumptions and stereotypes, improving communication skills and provide an enhanced awareness of the complexity of their patients’ lives [8]. Deep reflection has been associated with improved self-awareness, emotional learning, empowerment for critical thinking and greater ability to identify one’s learning needs [9].

**Figure 1:**
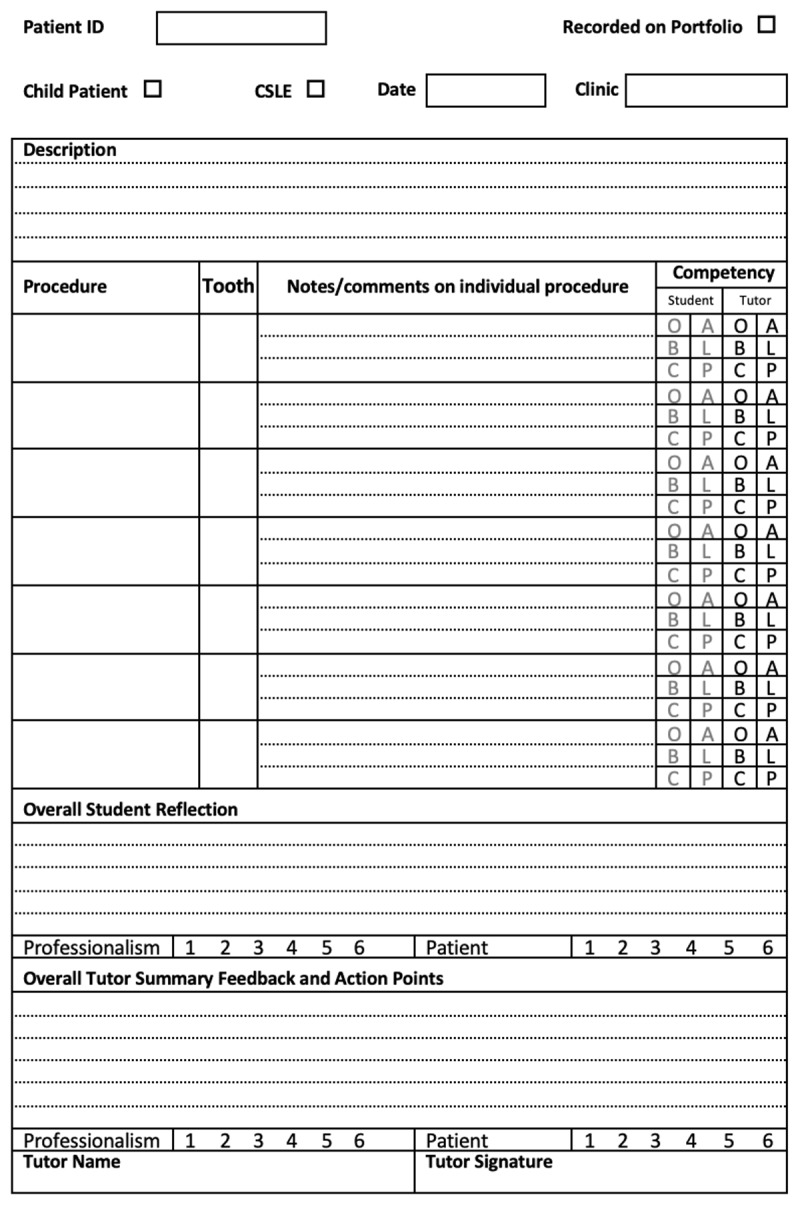
A sample page from the clinical logbook previously used for reflection. Reflection and assessment were facilitated together through ‘Competency’ grading and ‘Overall Student Reflection’. For definition; O, a student observed the session, A, a student assisted in the session, B a student performed as a beginner, L, a student performed as a learner, C, a student was competent, P, a student was proficient.

A recent literature review highlighted the need for a systematic overhaul of current methods of facilitating reflective practice in dentistry [10]. Furthermore, qualitative research by this team has demonstrated that the use of a written clinical logbook (Figure 1) to engage undergraduate dental students in deep reflection is unhelpful [11]. This study identified several barriers to reflective practice (Table 1) through the lens of the student, rather than educators, to whom these barriers had been invisible, justifying the need for a collaborative approach which involves students in the design and development of reflective practice.

**Table 1:** A summary of the key findings of “It’s like two stars and a wish”: A qualitative exploration of existing reflective practices used by undergraduate dental students [11].


UNDERSTANDING OF REFLECTION	

FINDINGS FROM QUALITATIVE RESEARCH	EXPLANATION OF PROBLEM

Reflection is a descriptive learning task	Reflection understood in very basic terms and seen as a way of seeing what had gone well and not

Reflection identifies improvements	Reflection valued as a way to identify positive aspects of practice and ensure that they could be developed

Reflection is a therapeutic process	Reflection enabled students to track growth in their confidence which was supported by constructive comments or reduced by focusing on negative aspects

**PREPARATION FOR REFLECTION**	

**FINDINGS FROM QUALITATIVE RESEARCH**	**EXPLANATION OF PROBLEM**

Informal process	Students could not recall any formal support in developing their reflective thinking and saw it as something that they ‘picked up on the way’

Uncertainty about logbook	Students were unclear on how the logbook should be used to reflect, with some thinking it was for documenting observations instead

Inadequate time	Reflecting using a logbook at the end of a clinical session led to a rushed unmeaningful experience with no time to prepare reflective thinking. This led to stress for students. Sometimes this led to a feedback conversation rather than a reflective one.

Safety aspects	Students were concerned that peers and other educators would be present in the time and environment dedicated to reflection, therefore leading to self-censorship and concealment of reflective feelings.

**IMPORTANCE OF LEARNING THROUGH EXPERIENCE**	

**FINDINGS FROM QUALITATIVE RESEARCH**	**EXPLANATION OF PROBLEM**

Improvement to patient care	Students understood the importance of reflective practice however saw it as a way to improve patient care rather than explore their personal thoughts and feelings

Educator/Student relationship	Students felt that opportunities for deep reflection were lost if educators provided superficial feedback

Peer feedback	Engaging in peer feedback was viewed as a helpful way to identify improvements which could be made to practice, in contrast to feedback from educators:

**SUGGESTIONS FOR DEVELOPMENT**	

**FINDINGS FROM QUALITATIVE RESEARCH**	**EXPLANATION OF PROBLEM**

Formal preparation	Students felt that a more forma and structured preparation for reflection would be beneficial

Optional activity	Students questioned the value of compulsory reflection on every patient contact that they had

Online options	Flexibility in the method of reflection, including online options and apps were suggested to better engage students in the reflective process


## Goal of Innovation

### Introduction to Co-Creation

The intended outcomes of deep reflection are closely related with and supportive of outcomes of co-creation. Co-creation is a “reciprocal process through which all participants have the opportunity to contribute equally, although not necessarily in the same ways, to curricular or pedagogical conceptualization, decision-making, implementation, investigation, or analysis” [12]. This approach has numerous reported benefits for students, staff and positive outcomes which are discussed in greater detail below.

There is increasing literature emerging outlining the value of co-creation through staff-student partnership in education, also referred to as ‘students as partners’ (SAP) [13]. Mercer-Mapstone identified 65 works between 2011 and 2015 that showed ‘a focus on partnership activities that are small scale, at the undergraduate level, extracurricular, and focused on teaching and learning enhancement [14]. They highlighted the need to move toward inclusive, partnered learning communities’ [14]. A very limited number (22%) of studies were applied in healthcare education, and these tended to be outcomes-based regarding co-creation projects, rather than process based [14]. Furthermore, co-creation has predominantly been reported in medical institutions in North America, therefore the application of this in dental education in a higher education institution within the United Kingdom is somewhat novel [13]. This study aimed to address this deficiency and provide experience-based insights on the process of undertaking co-creation, which can be applied in other areas of healthcare education.

A co-creation partnership facilitates both student and educator engagement in the research process. For students, this includes increased motivation and ownership for learning, increased sense of confidence and self-awareness, alongside a sense of belonging to university, discipline and community [14]. Students may also feel that they have greater agency over their education and develop skills that increase their employability [12]. Educators using co-creation might feel more inspired regarding education, clarifying their sense of self in their identity as an educator, and developing their understanding of learning and teaching by reconceptualising it as a collaborative process [12].

Co-creation has produced more ‘inclusive and relevant’ courses, with improved outcomes and attendance alongside better curricular design [15]. Other reported outcomes of co-creation include improved academic performance and quality of work, a more transparent learning process, a shift of student focus from grading to learning and the curriculum becoming more socially relevant [16].

Negative aspects associated with co-creation include a power imbalance, the inability to completely address systematic inequalities, and that often only a small number of students and staff are involved [17]. A lack of engagement, or even hostility and apathy from academics towards this approach has been described [18]. Further challenges include logistics, management of timelines and time commitments for success [13]. Future translation of the research, wider faculty and/or institutional support, building a trusting relationship, shifting mindset around traditional hierarchical roles and uni-disciplinary perspectives are also challenges [13].

There is no guidance on how to best facilitate deep reflection, and furthermore reflection is considered to be a wicked problem that cannot be solved by simply applying evidence, hence a novel, collaborative approach such as co-creation was indicated. The benefits of co-creation are clearly multifaceted for all involved, which makes it an attractive method for solving complex academic challenges such as this. Nonetheless, there is a paucity of evidence to inform the process of using co-creation to approach institutional academic challenges; a deficit that this study intended to address.

### Steps taken for Development and Implementation of innovation

This is a descriptive account of the process through which co-creation was applied to develop this intervention and draws on the informing evidence base (Table 2). Guidance from the Association for Medical Education in Europe (AMEE) on the application of co-creation for design and development of education has been applied where appropriate [19]. The lack of evidence in some aspects has resulted in trial and innovation by the research team to experiment in the application of co-creation to solve this wicked problem.

**Table 2:** A detailed description of the process of co-creation in developing a complex intervention to facilitate deep reflection for undergraduate dental students.


STEP1. FORMATION OF THE CO-CREATION TEAM	

DETAILS	RATIONALE

Recruitment of participants from the previous research study to join as co-creators [11]	Avoid power imbalance, between researchers and students [18]. Students had already volunteered solutions to the known problems associated with the previous method of reflecting. Inviting students to contribute to developing learning that they have experienced, can reflect on and that are ‘close to their heart’ raises their motivation and interest [19].

Core team confirmed, comprising five students and three researchers.	Five students volunteered. All were given the opportunity to join the co-creation team, to enable a distribution of workload and allow for discussion and collaboration within the student group. An experimental decision by the research team. The researchers were an external Professor of Nursing Education with expertise in reflective practice, a Clinical Lecturer in Paediatric Dentistry and a Specialty Registrar and Honorary Clinical Teacher in Paediatric Dentistry. This team had had a varied range of skills, relevant expertise and involvement in previous study on the topic.

Confirmation of time commitments, approximate deadlines and requirements of the project prior to committing to participating.	This stage is described as essential for success in the qualitative research synthesis of co-creation in higher education [13]. Enabled an informed commitment to participation and facilitated scheduling around students’ workload.

Involvement of other stakeholders within the institution, including members of the Dentistry Portfolio team and Student Support Lead.	These stakeholders would have significant involvement and interest in the developed intervention, and could benefit from the co-creation process [12]. Stakeholders were part of the wider team, rather than the core team, to ensure that the students were not uncomfortable with expressing opinions and that their voice was not diluted. This contributes to a sustainable and mutually beneficial intervention.

**STEP 2. DEFINITION OF ROLES, RESPONSIBILITIES AND COMMITMENTS**	

**DETAILS**	**RATIONALE**

Student roles: To co-design the intervention Resource design and development Peer teaching Opportunity for involvement in dissemination	The involvement of student voices and contributions as equals in publication assists in the endeavour for a more egalitarian approach to education [14].

Researcher roles: Arrange formal academic support for the student co-creators. Ensure that the intervention was appropriate and aligned with regulatory and University standards for undergraduate education, including developing briefs and reviewing works for the students. Act as an intermediary between the co-creators and faculty	To support open bidirectional feedback on the process and outcomes, connecting teachers and learners throughout [19].

**STEP 3. PREPARATION AND SUPPORT FOR STUDENT CO-CREATORS**	

**DETAILS**	**RATIONALE**

Guidance: Independent guidance provided by the Peer Assisted Learning (PAL) project based at the University of Sheffield. A supportive contact point for students throughout the project and provider of educational workshops on reflection and co-creation	Such arrangements can help in providing individual support, and discussing experiences and best practices in contributing to co-creation [19].

Recognition: Recognition for students’ extracurricular work in Teaching and Learning Resource Development was arranged through the Higher Education Achievement Report, a national recognition provided through the University of Sheffield for working collaboratively to plan and develop resources for teaching and learning.	The involvement of student voices and contributions as equals in publication assists in the endeavour for a more egalitarian approach to education [14].

Preparation: Students attended two workshops with the PAL team, where students had opportunities to share their thoughts through alternative communication means such as Google Jamboard (© Google, LLC),	Preparation was necessary to enable students to develop knowledge and reflective skills to empower them to meaningfully contribute. This also ensured that students were not working far beyond their expertise [19]. The opportunity to use alternative communication methods is recommended to afford co-creators more control and choice over how they share their thoughts [19].

Another workshop focused on exploring and addressing each barrier to reflective practice from the previous study, to outline a student proposal for the intervention. This was attended by a researcher (FC), and the Google Jamboards created during this session were saved for future reference.	The workshop acted as a refresher for the students, as some time had passed since they took part in the previous study.

**STEP 4. PLANNING AND DEVELOPMENT OF INTERVENTION**	

**DETAILS**	**RATIONALE**

The research team met to discuss the findings of the workshop attended by FC to discuss the feasibility of student suggestions and explore the development of a suitable proposal for the co-creation of educational resources and more flexible, secure methods of reflecting.	This was undertaken without students being present to not over burden them and to enable open discussion on the ideas from an academic perspective.

Proposal of a comprehensive intervention: Educational resources Development of a series of resources on the purpose and importance of reflection to address the disparity in the understanding of reflection, preparedness for reflection [11] Logbook changes Change from ‘reflection’ to ‘learning points’ allowed students to identify and record areas for improvement with clarity, acknowledging positive functions of the logbook reported by students to identify points for future learning and facilitate communication with educators [11] Changes to reflective process Students could reflect creatively in their own space and time in any way which felt appropriate for them, thus enabling the therapeutic process of reflection as previously described, by removing the time pressures of compulsory reflection at the end of a clinical session which had led to a rushed and unmeaningful experience [11]. Creation of an online environment for students to upload one reflective piece per clinical attachment and set their own privacy permissions to address safety concerns which could result in self-censorship and concealment of reflective feelings [11].	

Meeting 1 Discussed the content and delivery of each session, including videos, slideshows and audio commentary. Students were given autonomy in the development of the sessions. Meeting 2 and 3 Review of resources, with peer and researcher feedback. Additional meetings Meeting 4 The final educational resources were reviewed in by the student co-creators and FC.	This researcher was not involved in any form of assessment for co-creators, which is best practice and negates power imbalance [19]. Allowed students to bring any challenges meetings and work through them with peer discussion.

All workshops and meetings were undertaken online using Google Meet (© Google, LLC)	Necessary due to the ongoing COVID-19 pandemic

Resources reviewed by core co-creation team and wider stakeholders within the Dental School. Regular discussions with other stakeholders and the core researchers through email correspondence and six online meetings to achieve a mutually agreeable intervention. Key themes in negotiation were; time and resource requirements of a systematic overhaul in reflective practice, information governance and ensuring compliance with regulatory body requirements.	This allowed consideration of the practicalities, feasibility and acceptability of the proposed intervention that may not have been anticipated by core research team members in the planning stage. Students were not involved in the negotiation on the implementation of the intervention to avoid any power imbalance.

Secure online reflective environment was developed by IT colleagues to meet a brief set by the research team. Every aspect of this was reviewed by the students through email communication and undertaking test runs to review the software, including the fonts, colours and content. Students contributed links to helpful resources for undertaking reflection to this environment.	Researchers anticipated that the active involvement of student co-creators in this way would ensure that the design would be user-friendly, easily accessible and contain relevant information with clear security.


Ethical approval was gained from the University of Sheffield (Reference:038563).

Continual dialogue between the team ensured that the intervention was ready to launch within six months. Once all members of the team were satisfied, the launch was scheduled to coincide with the new academic year. The proposal was to be initially piloted within Paediatric Dentistry for third year undergraduate dental students only to allow for review and reform.

### Evaluation of Innovation

Educational resources for students and educators on reflective practice formed the primary output of the co-creation work. Online resources were chosen as a sustainable and flexible option that students could refer back to at any point. Six presentations were recorded and stored on Blackboard (©Blackboard Inc. 2022), a digital learning environment used throughout the University of Sheffield, with links to useful resources. Presentation topics were determined by the wider team and learning outcomes were developed by the core team. This included an introduction to reflective practice, the changes that were being implemented and expectations for students and educators for reflection. Student co-creators presented personal examples of creative methods of reflection within this. The resources were designed and produced by the co-creators, with support but minimal intervention from the core and wider team. Deadlines for this project were clearly defined but not rigid, following discussion led by the student co-creators to accommodate their studies. Clear outlining of roles, responsibilities and deadlines was valued by all members of the co-creation team. The co-creators were engaged and innovative, for example producing and including video recordings.

The secure online reflective environment (Reflection Portal) was embedded within Blackboard, and was designed to be flexible, to allow students to engage through more creative means, at a convenient time. This allowed the student to reflect when they felt ready and safe to do so, which may have been some time after the clinical situation. Reflective pieces could be uploaded in any format, including written, video, audio and photo files, allowing students to use an approach that suited their own reflective style. The portal was private, yet students could choose to share this with a member of staff if they wished. This addressed concerns with safety when reflecting using a logbook that is completed and stored on an open clinic.

A recommendation was made for each student to provide one reflective piece per clinical attachment in Paediatric Dentistry with no requirement for reflection in the logbook. Following discussions with the wider team and stakeholders, it was agreed that this approach would satisfy UK regulatory body requirements (GDC), which state in their Standards for Education that students provide ‘evidence of reflection’ [7]. This guidance is not prescriptive regarding the method of reflection [7]. Engagement with the reflective process was evaluated by the presence of a reflective piece accompanied by a short commentary. This acknowledged that reflection is a personal process, and hence not something that requires grading. Assessment of reflective pieces may shift the focus from reflection to assessment criteria and lead to self-censorship [20]. Students had the opportunity to discuss their reflection with a staff member if they wished to provide pastoral support in handling emotions that might surface through the reflective process.

## Critical Reflection

### Opportunities Associated with Co-creation

Co-creation has allowed for the development of a collaborative and respectful relationship between researchers and students, which all members benefitted from. This method is framed as a relational process rather than simply an outcome driven form of student engagement [12]. Success was facilitated by clearly outlining roles and responsibilities to all parties prior to commencing. This is essential for developing a genuine and organic partnership and is recommended for successfully applying co-creation in healthcare education [13].

The intervention and resources are community property for the co-creation team. This has assisted in compelling institutional commitment that would almost certainly not exist if the student co-creators were not the pioneers and developers. It is hard to ignore solutions to pedagogical problems highlighted and developed by service users.

The number of student co-creators was greater than planned, it has been suggested that this may contribute to a power imbalance [18]. Contrary to this the team found benefits of having five student co-creators and two researchers including peer support for students throughout the process, greater distribution of workload, and increased opportunity for collaboration. More voices gave more opportunity for sharing different experiences and perspectives, which co-creation has been credited with [12].

A recommendation for successful application of co-creation in healthcare education is flexibility from all partners such as with deadlines and adapting to inevitable change. Co-creation is a fluid process and a rigid structure would set one up for failure. Flexibility links to reflective practice itself, as it allows for more meaningful reflection.

The student co-creators were extremely motivated, whilst undertaking an intense healthcare course and examinations during a pandemic. Their confidence has increased with the co-creation process, from stating that they did not know how to reflect at all in previous work, to becoming those teaching their peers how to reflect [11]. Student co-creators have disseminated their work to wider audiences at conferences and through writing blogs. Their motivation and commitment demonstrate the value that they found in both co-creation and reflective practice. This illustrates the benefits of co-creation discussed in the literature such as confidence, investment, engagement and sense of scholarly belonging for students [14].

## Challenges associated with Co-creation

### Institutional Engagement

Institutional support is necessary in ensuring an effective and sustainable outcome [18]. The co-creation team had initially planned a systematic overhaul of reflective practices within the department and wider School; however, this has not yet been entirely achieved. The process of transitioning from the clinical logbook for reflective purposes to using the new intervention only, is still underway. This move has been more gradual than initially anticipated. It is understandable that there is some caution in engaging in radical curricular overhaul. This has commonly been reported in the literature alongside greater issues of low academic staff engagement, apathy, and even hostility to the approach of curricular development [18].

Establishing and maintaining institutional commitment and engagement has been challenging perhaps due to a lack of face-to-face contact with staff due to working from home and communicating mainly through videoconferencing due to the pandemic. There has been little to no opportunity for corridor communications and championing the project through physical presence. This particular group of teaching staff have also reported being affected by time pressures and heavy workloads. Other possible factors such as staff burnout following working through the pandemic, a lack of familiarity with the new intervention, a perceived lack of time, and strains on institution-staff relationships with ongoing dispute and industrial action regarding terms of employment within the UK may have added further institutional pressure in engaging in immediate change.

### Associated Sustainability Issues

Sustainability should be included as a key theme throughout co-creation. Online resources that could remain within the online learning platform were chosen and developed to ensure sustainability. This gave the student co-creators a legacy that could help with valuing their contribution. However, once the current students have graduated and are no longer champions for the project, momentum to keep the intervention going could decrease. This brings the risk of falling back to ‘what we have always done’. Maintaining this intervention is important because of the negative impacts that stresses of learning in a healthcare setting can have on student’s mental wellbeing [21].

Sustainability has been described as an issue with co-creation generally. When partnerships were developed as part of the formal curriculum, they were more likely to be ongoing in some form [13]. Projects that were more sustainable were those undertaken in whole programmes, that were committed to educational reform or involved staff who viewed their curriculum and teaching practices in uniquely relational ways [22]. Therefore, this raises the question of whether this intervention would have been more successful as an initial whole programme reform, rather than one undertaken in one dental discipline within one dental school.

## Discussion

This study has demonstrated that reflective practice is a valued and integral aspect of clinical education. Within healthcare education, reflection is often rendered ineffective with a rigid and reductionist approach such as reflection with assessment, as a competency and through portfolios. This study has shown that when reflection is respected as a whole, independent and integral part of learning then meaningful exploration of emotions and access to deep learning can occur.

### Strengths and Limitations

This study has several strengths which include the application and description of a novel method of applying co-creation in a healthcare education setting to address academic challenges. The detail and experience discussed provide valuable and unique insight into the practicalities of undertaking co-creation in similar situations. This acknowledges the expertise of students as service users.

This study has potential limitations that include the implementation of an intervention for third year undergraduate dental students in one U.K Dental School that was not part of the formal curriculum. The application of the method of using co-creation and intervention cannot be generalised. This is a subjective paper based on the experience of the researchers therefore cannot represent the views of the institution and wider team on the success of co-creation in this setting.

### Future Research

Future research plans include a mixed-methods evaluation of students’ experiences of the intervention, using Google forms, alongside a review of engagement with the new resources. The student co-creators will have the opportunity to be involved in this.

## Conclusion

This paper demonstrates the successful co-creation and implementation of a comprehensive intervention with undergraduate dental students to improve meaningful reflective practice. Despite posing some challenges, this process has generated numerous benefits for students, researchers and educators and is recommended for tackling similar problems in wider healthcare education.
